# Importance of Considering the Duplex Collecting System During Routine Endourological Procedures: A Case Report

**DOI:** 10.7759/cureus.100345

**Published:** 2025-12-29

**Authors:** Mahdi Mottaghi, Pranay Manda, Ernest A Morton, Mohammad Hajiha

**Affiliations:** 1 Department of Urology, Duke University Medical Center, Durham, USA; 2 Department of Urology, Emory University School of Medicine, Atlanta, USA

**Keywords:** bifid or double ureter, nephrolithiasis, ureteral obstruction, ureteral stents, urogenital abnormalities

## Abstract

We report the case of a 58-year-old woman who initially presented to the emergency department with intermittent nausea, vomiting, and left upper quadrant abdominal pain for six days. As part of the abdominal pain workup in the emergency department, contrast-enhanced abdominopelvic CT and laboratory assessment were performed, which were consistent with pyelonephritis secondary to a distal ureteral stone. Interestingly, while the imaging failed to detect any significant anatomic variation, a duplicated collecting system was diagnosed during ureteral stent placement. Recognition of this anomaly was crucial for successful patient management. Awareness of anatomic variants is essential during endourological procedures, as diagnostic imaging may not reliably detect them.

## Introduction

Renal collecting system duplication, also known as ureteral duplication (UD) or duplex kidney, occurs when a kidney contains two pyelocaliceal systems with a single or two distinct ureteral openings in the bladder [1]. This anomaly is present in approximately 0.6-1% of healthy adults, with higher prevalence (approximately 2-4%) among patients evaluated for urinary tract symptoms [2,3]. It has been shown that UD is relatively more common in females, and related genes are inherited in an autosomal dominant pattern with incomplete penetrance [4].

A duplex urinary system is a normal anatomical variant if both upper and lower portions function normally and cause no symptoms; however, common urological diseases such as stones, vesicoureteral reflux, urinary tract infections (UTIs), and ureteropelvic junction obstructions can still occur [1,5]. It is important for the surgeon to maintain awareness of UD during procedures to avoid complications. Here, we present a case of acute pyelonephritis in which UD was diagnosed during ureteral stent placement for an obstructive calculus. While this patient was managed successfully, missing UD can complicate evaluation and subsequent treatment [6], as the anomaly may not be visible on initial imaging.

## Case presentation

**Figure 1:**
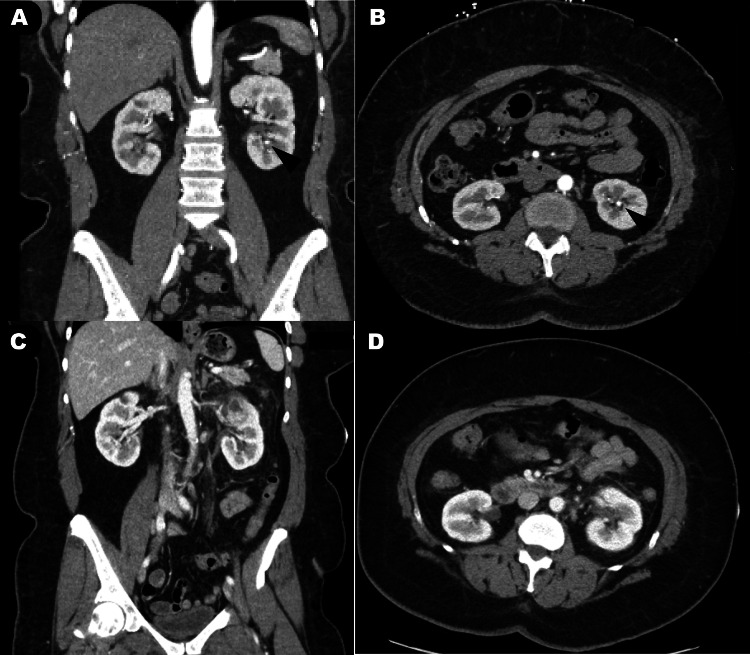
Abdominopelvic CT with contrast ordered by the emergency medicine team for abdominal pain workup. Contrast-enhanced CT imaging of the kidneys. Panels A (coronal) and B (axial) are from an abdominopelvic CT with contrast done a year earlier, and an incidental 5-mm stone was detected with no anatomic anomalies noted. Panels C (coronal) and D (axial) are from the imaging a day before the surgery, ordered by the emergency medicine team.

A 58-year-old woman presented to the emergency room with a chief complaint of intermittent nausea, vomiting, diarrhea, and abdominal pain that started six days prior. She attributed the symptoms to food poisoning but mentioned an episode of malodorous urine without dysuria one day before presentation. Past medical history was significant for diabetes, hypertension, systemic lupus erythematosus, and a prior history of a 5-mm left, inferior pole, kidney stone mentioned as an incidental finding on a contrast-enhanced abdominopelvic CT (Figures 1A, 1B) a year earlier for assessment of a possible aortic aneurysm; of note, no anatomical anomaly was noted in that report. On physical examination, she was found to be afebrile with stable vital signs and left costovertebral angle tenderness. Laboratory studies revealed a creatinine of 0.96 mg/dL, and urinalysis was significant for pyuria (Table 1) and hematuria (10 red blood cells/hpf). Urine culture collected in the emergency department came back positive for pansensitive *Klebsiella pneumoniae*. Contrast-enhanced CT (Figures 1C, 1D) obtained by the emergency department showed a 6-mm distal left ureteral stone with associated perinephric fat stranding, suggesting pyelonephritis. Given the presence of an obstructing stone in the setting of an acute infection, the patient was taken emergently to the operating room for urinary decompression via ureteral stent placement.

**Table 1:** The relevant laboratory values with the normal ranges at baseline, admission, and discharge. ^†^: Two months prior to admission; ^*^: Abnormal BUN = blood urea nitrogen; Hct = hematocrit; Hgb = hemoglobin; HPF = high‑power field (microscopy); LPF = low‑power field (microscopy); Plt = platelets; WBC = white blood cells

Test	Baseline^†^	Admission	Discharge	Normal range
Sodium	140	136	—	135–145 mmol/L
Potassium	4.1	4.0	—	3.5–5.0 mmol/L
Chloride	105	102	—	98–107 mmol/L
Bicarbonate	28	26	—	22–29 mmol/L
Anion gap	7 (L)	8	—	8–16 mmol/L
BUN	34 (H)	10	—	7–20 mg/dL
Creatinine	1.16	0.96	—	0.6–1.3 mg/dL
WBC	—	11.0 (H)	9.1	4.0–10.5 × 10³/µL
Hemoglobin	—	11.5	10.8 (L)	12–16 (F), 13.5–17.5 (M) g/dL
Hematocrit	—	37.0	36.1	36–46% (F), 41–53% (M)
Platelets	—	205	217	150–450 × 10³/µL
Urine analysis				
Specific gravity	—	1.026	—	1.003–1.030
pH	—	5.5	—	5.0–8.0
Protein (qualitative)	—	20 mg/dL	—	<30 mg/dL
Glucose	—	Negative	—	Negative
Ketones	—	Negative	—	Negative
Blood (qualitative)	—	Negative	—	Negative
Urobilinogen	—	4 mg/dL (*)	—	0.2–1.0 mg/dL
Nitrite	—	Positive (*)	—	Negative
Leukocyte esterase	—	Large (*)	—	Negative
Bilirubin	—	Negative	—	Negative
WBC/HPF	—	61 (*)	—	0–5 /HPF
RBC/HPF	—	10 (*)	—	0–3 /HPF
Epithelial cells/HPF	—	8 (*)	—	<5 /HPF
Mucus/LPF	—	4+ (*)	—	None–rare
Urine culture	—	>=100,000 CFU/mL; *Klebsiella pneumoniae* ssp *pneumoniae*	—	—

**Figure 2:**
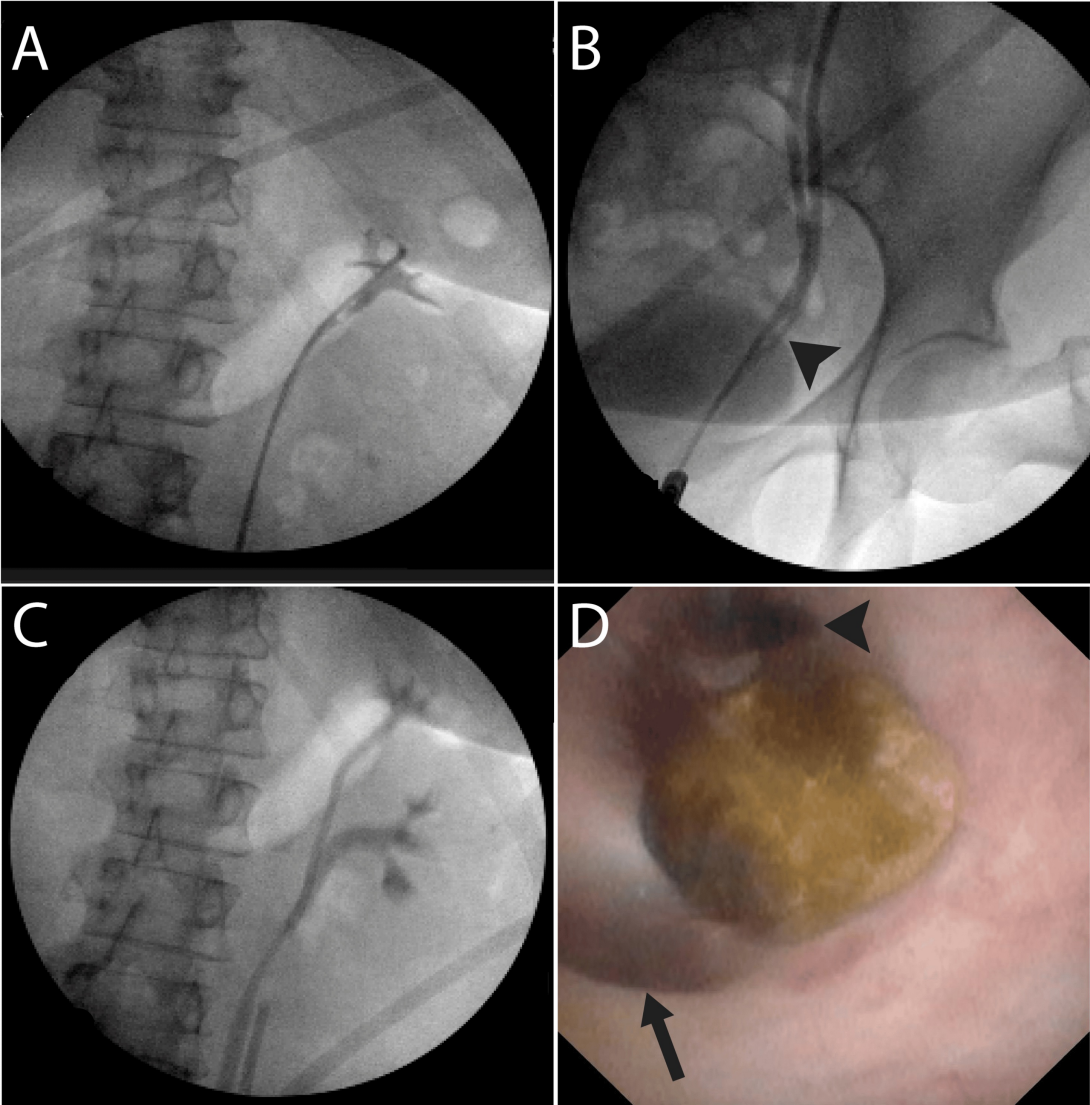
Ureteral stent placement procedure. (A) Hypoplastic renal pelvis. (B) Retrograde pyelography using minimal contrast, revealing a V‑shaped incomplete ureteral duplication. (C) Delayed to no drainage of contrast from the lower pole of the left kidney. (D) Impacted stone visualized under semirigid cystoscopy and extracted. Note the ureteral junction of the upper-pole ureter (arrow) and the lower-pole ureter (arrowhead).

During the procedure, the wire passed easily through the left ureter, and stent placement was confirmed in the renal pelvis with fluoroscopy; however, the renal pelvis appeared hypoplastic to the surgeon’s eyes. This finding, in addition to the lack of a hydronephrotic drip, raised concern for a possible duplex system and prompted further intraoperative assessment. Thus, a retrograde pyelogram (RGP) was performed despite it being a relative contraindication during an active UTI. To minimize the risk of infection spread, the RGP was performed (Figure 2A) after decompression of the calyceal system with only 4 mL of contrast, which revealed a V-shaped duplicated ureter (Figure 2B). The lower pole was mildly distended, and there was no contrast drainage from it (Figure 2C), suggesting that only the lower-pole ureter was obstructed. We then attempted to pass a wire into the lower-pole ureter with a Kumpe catheter; however, this was unsuccessful due to the acute angle of the branch point for the duplicated ureters. We then chose to utilize a semirigid ureteroscope with minimal irrigation to enter the ureteral orifice and visualize the duplication. The ureters were noted to join approximately 10 mm proximal to the orifice. The impacted stone was visible at the distal opening of the lower-pole ureter (Figure 2D). The distal ureter was patent, and the calculus was removed easily with a basket. A wire was placed through the semirigid scope into the lower pole ureter under direct visualization and exchanged for a 6-Fr double J ureteral stent. The patient was discharged on cefpodoxime postoperatively. The stent was removed two weeks later, with the patient being asymptomatic and fully recovered.

## Discussion

UD presents on a spectrum. It can be incomplete and present as a bifid, Y-shaped (mid-ureteral joining point), or V-shaped ureter (joining point near or within the bladder) [1]. A complete UD has two distinct ureteral orifices. In this scenario, the ureter from the lower pole travels similarly to a normal ureter of a single collecting system and inserts into the bladder. However, the ureter from the upper pole could attach to several anatomical locations, with the most common insertion site within the bladder in both females and males; thus, UD is most commonly asymptomatic. In such cases, the upper-pole ureter attaches medially and inferiorly to the inferior-pole ureter, which is known as the Weigert-Meyer rule [1]. In boys, the ectopic ureter from the upper pole can be inserted into the bladder, and less commonly into the prostatic urethra, ejaculatory ducts, seminal vesicles, or the vas deferens, all of which are proximal to the external urethral sphincter. In girls, however, the upper-pole ureter can insert into the bladder, urethra distal to the urethral sphincter, perineum, vagina, and, rarely, into the uterus and fallopian tubes. Thus, UD may present with constant wetness in female children [1].

As mentioned, most UD cases are asymptomatic, as both upper and lower poles maintain normal renal function. In patients with UD, the radiologic appearance of the kidney may appear longer than that of a kidney with a single collecting system; it may be overlooked [1]. Zheng et al. reported a similar case with UTI and ureteral obstruction, who was discharged home following stent placement in the upper moiety of the left kidney, similar to our case [6]. However, the patient was readmitted after two weeks when the diagnosis of UD was made. In the setting of an obstructing ureteral stone in a patient with a duplicated system, it is possible to stent the non-obstructed ureter and thus fail to surgically address the underlying issue. The risk of placing a stent in the non-obstructed ureter is increased when the anatomic variant is not properly identified before urinary decompression. This further signifies the importance of considering the mentioned anatomical variances in routine procedures.

The first main take-home message of the presented case is that maintaining alertness for potential duplication of the renal collecting system is crucial, as overlooking these anatomic variations and failure to adjust surgical techniques by the urologist or the interventional radiologist can significantly hinder patient management. For example, the presented patient had undergone several contrast-enhanced CT scans within the past two years of presentation for reasons other than urinary calculus, and none of these scans reported any clues to suggest UD.

The other important takeaway is that although RGP is relatively contraindicated during active UTI, when deemed necessary by the surgeon, minimal contrast should be injected to limit the rise in intrarenal pressure and decrease the risk of sepsis. We used only 4 mL of contrast in this case, and preoperative ceftriaxone with postoperative cefpodoxime to address the underlying UTI was sufficient for controlling possible infection spread.

The lower-pole ureter is usually the dominant drainage of a duplicated system, and stone impaction occurs more frequently in the lower moiety compared to the upper pole [5], as seen in this case.

## Conclusions

This case highlights the importance of recognizing UD during routine endourological procedures. Because routine imaging, such as contrast-enhanced CT scans, may fail to reveal the anomaly, surgeons need a high index of suspicion whenever intraoperative observations do not fit typical anatomy. This is vital in reducing avoidable errors and improving the overall safety and success of patient care.
